# Glass Brushes for Applying Fluid Caustics

**Published:** 1855-09

**Authors:** 


*Glass Brushes for Applying Fluid Caustics.*—It is desirable, in the use of any of the mineral acids as escharotics, that their strength should not be diminished by the employment of any material susceptible of being charred. A serious objection, therefore, lies against the use of wood, cotton-wood, lint, &c., all of which have been recommended for that purpose. Glass is by far the best material, being at once durable, cleanly, easy of use, and quite insusceptible of the action of the fluid. A glass rod, rounded at one end, and drawn to a fine point at the other, may be made to serve most purposes, one or the other extremity being employed according as it is wished to apply the acid over a large or small extent of surface. A few brushes, of different sizes, made of spun glass, are, however, yet more convenient. Those sold in the shops are much too large for most of the purposes mentioned in the above notice of the uses of the acid nitrate, and we have seen none which would exactly meet the required conditions, excepting those in use at the hospital for Skin Diseases. With a little glass tubing, of the thickness of a quill, a skein of spun glass, and a little sealing-wax, they may be inexpensively made by any one of ordinary ingenuity. The brush part is first made by uniting together with sealing-wax a tuft of the spun material, and is then introduced into the end of a tube, which has been either flattened out or brought nearly to point while heated. A little additional heat easily fixes the tuft in position, and by scissors it may then cut down to the requisite size.—*Med. Times and Gaz.*, January 6th, 1855.

*Extripation of the Uterus.*—Dr. Reiche states that he has extripated the entire uterus seven times; and in all cases the result was fatal. He, however, advocates the operation in cases of cancer confined to the organ. He then describes the method of operating; this presents nothing calling for analysis. Partial extripation he represents as a painful operation, but one free from danger. It is indicated in all degenerations limited to the neck of the uterus —*Brit. and For. Med. Chirurg. Rev.* April, 1854, from *Deutshe Klin.* 43. 1854.

